# Clinical Characteristics and Comparative Proteomics Analysis of COVID-19-Related Atrioventricular Block

**DOI:** 10.31083/j.rcm2506195

**Published:** 2024-05-28

**Authors:** Yuan Gao, Zhongli Chen, Sijin Wu, Ruohan Chen, Yan Dai, Shu Zhang, Keping Chen

**Affiliations:** ^1^State Key Laboratory of Cardiovascular Disease, Arrhythmia Center, Fuwai Hospital, National Center for Cardiovascular Diseases, Chinese Academy of Medical Sciences and Peking Union Medical College, 100037 Beijing, China

**Keywords:** COVID-19, atrioventricular block, comparative proteomics analysis

## Abstract

**Background:**

Atrioventricular block (AVB) is thought to be a rare cardiovascular complication of the coronavirus disease 2019 (COVID-19), though limited data are available beyond case reports. We aim to describe the baseline characteristics, proteomics profile, and outcomes for patients with COVID-19-related AVB.

**Methods:**

We prospectively recruited patients diagnosed with COVID-19-related AVB between November 2022 and March, 2023. Inclusion criteria were hospitalization for COVID-19 with the diagnosis of AVB. A total of 24 patients diagnosed with COVID-19 without AVB were recruited for control. We analyzed patient characteristics and outcomes and performed a comparative proteomics analysis on plasma samples of those patients and controls.

**Results:**

A total of 17 patients diagnosed with COVID-19-related AVB and 24 individuals diagnosed with COVID-19 infection without AVB were included. Among patients with COVID-19-related AVB, the proportion of concurrent pneumonia was significantly higher than controls (7/17 versus 2/24, *p* < 0.05). All 17 AVB patients (9 of permanent AVB, 8 of paroxysmal AVB) received permanent pacemaker implantation. No procedural-related complication occurred. In laboratory tests, the level of biomarkers indicating myocardial damage were substantially higher than controls, including high-sensitivity cardiac troponin-I (median 0.005 versus 0.002 ng/mL, *p* < 0.05), myoglobulin (median 39.0 versus 27.6 ng/mL, *p* < 0.05), and MB isoenzyme of creatine kinase (median 1.2 versus 0.8 U/L, *p* < 0.05). The level of N-terminal pro-b-type natriuretic peptide (median 241.0 versus 33.5 pg/mL, *p* < 0.05), C-reactive protein (median 4.8 versus 2.0 mg/L, *p* < 0.05), D-dimer (median 1.2 versus 0.2 µg/mL, *p* < 0.05), left ventricular end-diastolic diameter (median 49.3 versus 45.7 mm, *p* < 0.05) in patients with COVID-19-related AVB were significantly higher than controls. The level of albumin (median 41.9 versus 44.5 g/L, *p* < 0.05) was significantly lower than controls. In comparative proteomics analysis, we identified 397 human proteins. Several significantly altered plasma proteins related to inflammatory response (Serum amyloid A protein, C-reactive protein, Protein Adenosine 5’-monophosphate-activated protein kinase (AMPK), Alpha-2-macroglobulin), complement and coagulation cascades (Tetranectin, haptoglobin), and immune response (Neutrophil defensin 3, Fibrinogen beta chain) may contribute to the pathogenesis of COVID-19-related AVB.

**Conclusions:**

Patients with COVID-19-related AVB are more prone to have myocardial damage and concurrent pneumonia. Through laboratory tests and comparative proteomics analysis, we identified several differential expressed proteins (Serum amyloid A protein, Tetranectin, Neutrophil defensin 3) releated to the inflammatory response, complement and coagulation cascades, and immune response, which provides evidence of potential biomarkers and sheds light on the pathogenesis of COVID-19-related AVB.

## 1. Introduction

Severe acute respiratory syndrome coronavirus 2 (SARS-CoV-2) infection or coronavirus disease 2019 (COVID-19) has caused a worldwide pandemic that continues to be relevant globally. Alongside the respiratory system, an arrhythmogenic effect of COVID-19 has been recognized. Tachyarrhythmias such as atrial tachycardia, sinus tachycardia, and ventricular arrhythmias are documented as the most frequent dysrhythmias [1, 2, 3]. Though bradyarrhythmia presenting as atrioventricular block (AVB) and sinus bradycardia are less frequently reported, they are thought to have a worse prognosis [4, 5].

Although increasing amounts of data have reported the incidence of COVID-19-related AVB, however, the clinical characteristics, classification, and outcomes have not been well summarized. Moreover, several hypotheses as cytokine storms, direct viral damage, hypoxia, and immune disorders, have been thought to influence the cardiac conduction system, but the concrete pathogenesis of COVID-19-related AVB remains unknown [6, 7, 8, 9, 10].

In this study, we recruited 17 COVID-19-related AVB patients and summarized their clinical characteristics and outcomes. We hypothesized COVID-19 induces molecular changes that can be detected in the patient’s sera and used mass spectrometry assays for data-independent acquisition to comparatively analyze the proteomics profile of COVID-19-related AVB patients and several control groups.

## 2. Materials and Methods

### 2.1 Study Population

**Fig. 1.**
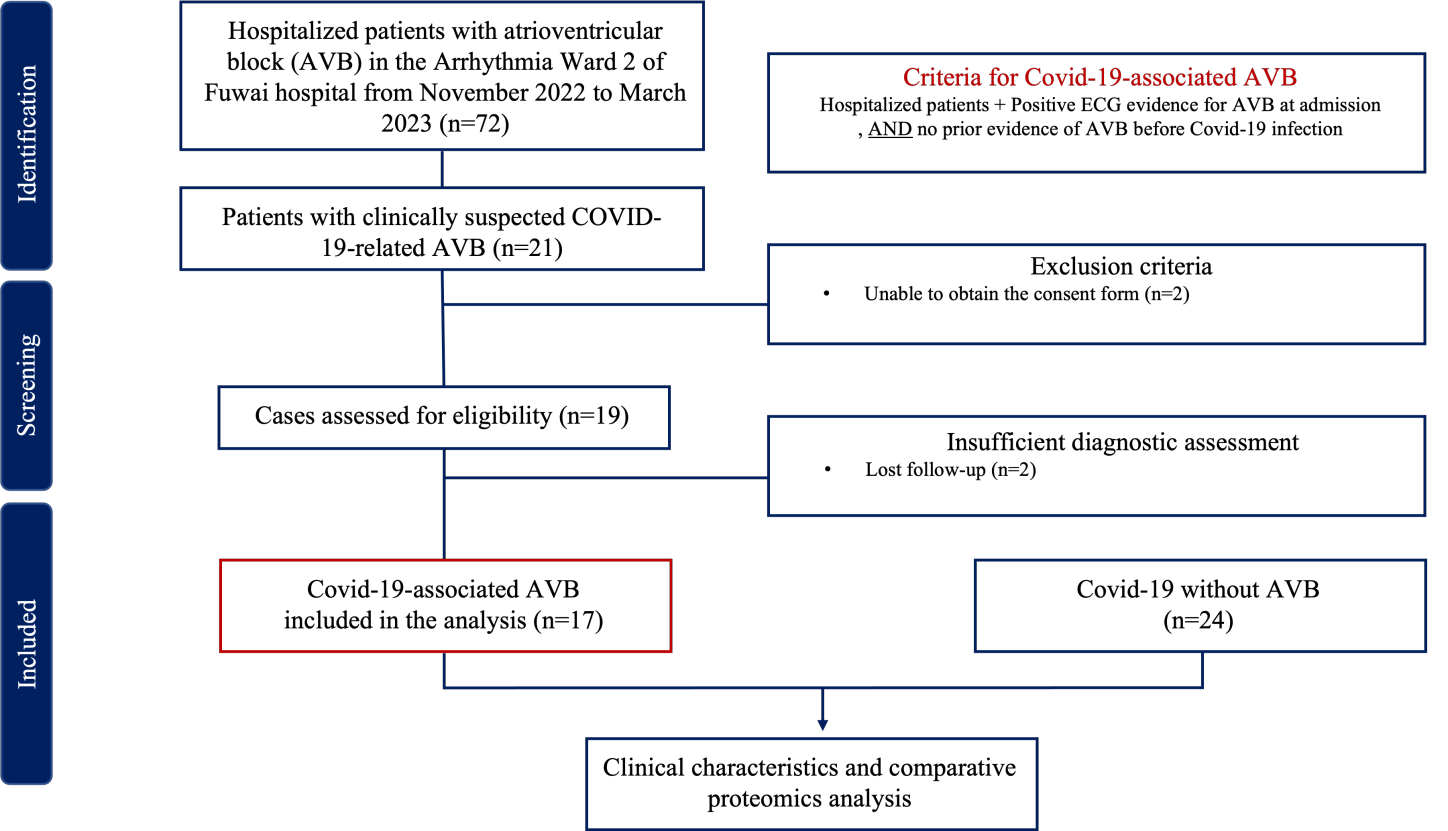
**Flow chart of inclusion and exclusion criteria.** ECG, electrocardiogram; AVB, atrioventricular block; COVID-19, coronavirus disease 2019.

This is a single-center, prospective, observational study. A total of 17 patients diagnosed with COVID-19-related AVB, and 24 individuals diagnosed with COVID-19 without AVB were recruited from November 2022 to March 2023. The inclusion and exclusion criteria are shown in Fig. 1. This study complied with the Declaration of Helsinki and was approved by the ethics committee of Fuwai Hospital Chinese Academy of Medical Sciences. We obtained informed consent from all participants. All COVID-19-related AVB patients enrolled in this study have to fulfilled the inclusion criteria: (1) Definite SARS-CoV-2 infection evidenced by positive RT-PCR nasopharyngeal swab or rapid antigen detection test. (2) No prior evidence of AVB from resting electrocardiogram and/or ambulatory electrocardiogram before the COVID-19 infection. Among the suspected cases of COVID-19-related AVB, cases were excluded for the following reason: (1) Unable to obtain the consent form.

### 2.2 Data Collection and Definition

Medical history, clinical characteristics, laboratory tests, and outcomes were obtained from in-hospital medical records. A transthoracic echocardiogram was performed according to the latest guidelines. Left ventricular ejection fraction (LVEF) was measured by Simpson’s biplane technique. Cardiac magnetic resonance imaging (MRI) evaluation included T1 and T2 mapping, late gadolinium enhancement (LGE), and conventional cine. For patients who were in need of a permanent pacemaker, sensed and paced atrioventricular intervals were programmed as 170 and 200 ms at follow-up.

### 2.3 Plasma Sample Preparation and Fractionation for Data Dependent Acquisition (DDA) Library Generation

Proteins of serum pools were separated using the Human 14 Multiple Affinity Removal System Column (LOT-51886560, Agilent Technologies, Beijing, China) following the manufacturer’s protocol [11]. The low and high-abundance proteins were collected respectively, desalinated, and concentrated using an ultrafiltration tube. Then we added SDT buffer (100 mM Tris-HCl pH 7.6, 4% SDS), boiled for 15 min and centrifuged at 14,000 g for 20 min. We used the BCA Protein Assay Kit (LOT-5000001, Bio-Rad, Berkeley, CA, USA) to quantify the supernatant. The sample was then stored at –80 °C.

### 2.4 Filter-Aided Sample Preparation Digestion Procedure

We added the DTT (LOT-161-0404, Bio-Rad, Berkeley, CA, USA) to each sample and mixed it. Iodoaceamide (LOT-163-2109, Bio-Rad, Berkeley, CA, USA) was added to block reduced cysteine residues and then incubated for 30 min in darkness. Next, the samples were filtrated and washed with 100 µL UA buffer and 100 µL 25 mM ${\mathrm{NH}}_{4}$${\mathrm{HCO}}_{3}$ buffer. We added trypsin to the samples and incubated them at 37 °C overnight.

Then each sample was desalted on C18 Cartridges (LOT-Empore™ C18, Sigma, St. Louis, MO, USA), concentrated, and reconstituted in formic acid. The peptide was measured by 280 nm UV light. Calibration peptides were spiked into the sample for DIA experiments.

### 2.5 Mass Spectrometry Assay for Data-Independent Acquisition (DIA)

We used a Q-Exactive HF-X mass spectrometer to analyze each sample by the Easy-nLC 1200 chromatography system in the data-independent acquisition (DIA) mode. Each DIA cycle contained one full MS–SIM scan, and 44 DIA scans covered a mass range of 350–1800 m/z.

### 2.6 Mass Spectrometry Data Analysis

The FASTA sequence database was searched with ${\mathrm{Spectronaut}}^{\text{TM}}$ software (Version 17.0, Biognosys, Newton, MA, USA) for DDA library data. We downloaded the database at http://www.uniprot.org and added iRT peptides sequence. The identification of protein data was determined by a false discovery rate of ≤1%. We used ${\mathrm{Spectronaut}}^{\text{TM}}$ software to search the constructed library for analyzing DIA data. All data were filtered by a false discovery rate of ≤1%.

### 2.7 Statistical Analysis

We use the Anderson-Darling test and Shapiro-Wilk test to evaluate the normality of continuous variables. Continuous variables that passed the normality test were reported as mean and standard deviation. Continuous variables that failed the normality test were recorded as median and quartile 1 (Q1) to quartile 3 (Q3). The unpaired *t*-test and Mann-Whitney test were used to analyze the continuous variables. We use Fisher exact test to compare the categorical variables. All statistical analyses were two-tailed. *p* value < 0.05 was considered statistically significant.

## 3. Results

### 3.1 Baseline Characteristics and Laboratory Profiles of Study Population

Baseline characteristics of all 17 COVID-19-related AVB patients are listed in Table 1. The mean age was 59.9 years old, 29.4% of the patients were female, and all had received a COVID-19 vaccination before the onset of AVB. The diagnosis of SARS-CoV-2 infection was confirmed by a positive RT-PCR nasopharyngeal swab (76.5%) or rapid antigen detection test (82.4%). Eleven (64.7%) patients had intermittent cough, and 13 (76.5%) patients had fever during COVID-19. Noticeably, all 17 patients had palpitation, 8 patients (47.1%) had syncope onset, 5 patients (29.4%) complained of chest pain. Nine patients (52.9%) had permanent AVB, and 8 patients (47.1%) had paroxysmal AVB. Seven patients (41.2%) had AVB with pneumonia. The median length of stay was 7.0 days (Q1–Q3: 6.0–9.0) with a maximum hospital stay of 15 days.

**Table 1.** **Baseline characteristics of study population**.

		All	AVB with COVID-19	COVID-19 without AVB	*p* value
Overall		41	17	24	
Demographics					
	Age, years, mean ± SD	56.8 ± 1.9	59.9 ± 15.2	54.7 ± 8.7	0.17
	Female sex, n (%)	14 (34.1)	5 (29.4)	9 (37.5)	0.74
	BMI, kg/$m^{2}$, mean ± SD	24.6 ± 3.3	23.7 ± 3.3	25.3 ± 3.2	0.13
	Hypertension, n (%)	14 (34.1)	11 (64.7)	3 (12.5)	<0.01*
	Diabetes, n (%)	3 (7.3)	3 (17.6)	0 (0.0)	0.12
	Coronary artery disease, n (%)	4 (9.8)	4 (23.5)	0 (0.0)	0.06
	Current or previous smoker, n (%)	14 (34.1)	7 (41.2)	7 (29.2)	0.51
	Current or previous cancer, n (%)	3 (7.3)	2 (11.8)	1 (4.2)	0.56
	Autoimmune disorder, n (%)	1 (2.4)	1 (5.9)	0 (0.0)	0.41
PR interval before COVID-19 infection, ms, mean ± SD		135.0 ± 31.0	141.5 ± 29.6	135.6 ± 27.7	0.52
ECG on admission					
	Third-Degree AVB, n (%)	14 (34.1)	14 (82.4)	-	
	Permanent AVB, n (%)	9 (22.0)	9 (52.9)	-	
Pneumonia on CT scan, n (%)		9 (22.0)	7 (41.2)	2 (8.3)	0.02*
Laboratory tests on admission					
	WBC, ×${\mathrm{10}}^{9}$/L, median (Q1–Q3)	6.0 (4.7–7.3)	6.8 (5.2–8.9)	5.7 (4.3–6.9)	0.01*
Lymphocyte count, ×${\mathrm{10}}^{9}$/L, median (Q1–Q3)		1.6 (1.3–2.1)	1.5 (1.2–1.8)	1.8 (1.4–2.3)	0.09
	hs-cTnI, ng/mL, median (Q1–Q3)	0.002 (0.002–0.007)	0.005 (0.003–0.027)	0.002 (0.002–0.003)	<0.01*
	NT-pro BNP, pg/mL, median (Q1–Q3)	67.5 (20.4–228.0)	241.0 (122.5–1684.0)	33.5 (17.8–66.5)	<0.01*
	MYO, ng/mL, median (Q1–Q3)	31.2 (22.6–50.0)	39.0 (29.4–76.0)	27.6 (20.4–43.4)	0.04*
	CK-MB, U/L, median (Q1–Q3)	0.8 (0.6–1.3)	1.2 (0.6–1.5)	0.8 (0.5–1.2)	0.03*
	CRP, mg/L, median (Q1–Q3)	2.6 (1.8–5.0)	4.8 (2.7–6.7)	2.0 (1.6–3.4)	<0.01*
	D-dimer, µg/mL, median (Q1–Q3)	0.3 (0.2–1.2)	1.2 (0.2–3.7)	0.2 (0.2–0.3)	<0.01*
	ALT, IU/L, median (Q1–Q3)	22.0 (15.5–34.0)	21.0 (14.5–31.5)	27.0 (15.5–38.5)	0.28
	Creatinine, mg/dL, median (Q1–Q3)	72.9 (59.8–84.7)	69.4 (61.4–93.2)	72.9 (60.8–81.2)	0.63
	Albumin, g/L, median (Q1–Q3)	45.0 (39.9–46.4)	41.9 (38.0–45.3)	45.5 (44.5–47.6)	<0.01*
Echocardiography on admission					
	LVEDD, mm, mean ± SD	47.2 ± 4.7	49.3 ± 4.3	45.7 ± 4.5	0.01*
	LVEF, %, mean ± SD	64.7 ± 6.1	63.7 ± 7.3	65.4 ± 5.1	0.37

AVB, atrioventricular block; BMI, body mass index; WBC, white blood cell; hs-cTnI, high-sensitivity cardiac troponin I; NT-pro BNP, N-terminal pro b-type natriuretic peptide; MYO, myoglobulin; CK-MB, MB isoenzyme of creatine kinase; CRP, C-reactive protein; ALT, alanine transaminase; LVEDD, left ventricular end diastolic diameter; LVEF, left ventricular ejection fraction. * indicates statistically significant; COVID-19, coronavirus disease 2019; SD, standard deviation; ECG, electrocardiogram; CT, computed tomography.

In laboratory tests, the level of high-sensitivity cardiac troponin-I (median 0.005 versus 0.002 ng/mL, *p* < 0.05), myoglobulin (median 39.0 versus 27.6 ng/mL, *p* < 0.05), and MB isoenzyme of creatine kinase (median 1.2 versus 0.8 U/L, *p* < 0.05) were significantly higher than controls. The level of N-terminal pro-b-type natriuretic peptide (median 241.0 versus 33.5 pg/mL, *p* < 0.05), C-reactive protein (median 4.8 versus 2.0 mg/L, *p* < 0.05), D-dimer (median 1.2 versus 0.2 µg/mL, *p* < 0.05) in patients with COVID-19-related AVB were substantially higher than controls. The details of characteristics of 9 COVID-19-related AVB patients and controls for proteomics analysis are listed in Table 2.

**Table 2.** **Baseline characteristics of individuals recruited for comparative proteomics analysis**.

		All	AVB with COVID-19	COVID-19 without AVB	*p* value
Overall		19	9	10	
Demographics					
	Age, years, mean ± SD	51.0 ± 16.7	55.7 ± 18.3	46.8 ± 14.8	0.26
	Female sex, n (%)	9 (47.4)	3 (33.3)	6 (60.0)	0.37
	BMI, kg/$m^{2}$, mean ± SD	25.6 ± 3.8	25.1 ± 3.6	26.1 ± 4.1	0.58
	Hypertension, n (%)	7 (36.8)	5 (55.6)	2 (20.0)	
	Diabetes, n (%)	3 (15.8)	3 (33.3)	0 (0.0)	0.09
	Coronary artery disease, n (%)	1 (5.3)	1 (11.1)	0 (0.0)	0.47
	Current or previous smoker, n (%)	5 (26.3)	3 (33.3)	2 (20.0)	0.63
	Current or previous cancer, n (%)	3 (15.8)	2 (22.2)	1 (10.0)	0.58
	Autoimmune disorder, n (%)	1 (5.3)	1 (11.1)	0 (0.0)	0.47
ECG on admission					
	Third-Degree AVB, n (%)	9 (47.4)	9 (100.0)	-	
	Permanent AVB, n (%)	6 (31.6)	6 (66.7)	-	
Laboratory tests on admission					
	WBC, ×${\mathrm{10}}^{9}$/L, median (Q1–Q3)	6.3 (5.3–7.4)	7.4 (5.2–9.4)	6.1 (5.0–7.0)	0.18
Lymphocyte count, ×${\mathrm{10}}^{9}$/L, median (Q1–Q3)		1.5 (1.2–2.2)	1.4 (0.9–2.1)	1.5 (1.3–2.4)	0.56
	hs-cTnI, ng/mL, median (Q1–Q3)	0.002 (0.002–0.009)	0.005 (0.002–0.027)	0.002 (0.002–0.005)	0.12
	NT-pro BNP, pg/mL, median (Q1–Q3)	96.1 (18.2–256.0)	206.0 (116.0–1098.0)	36.2 (16.9–75.3)	<0.01*
	MYO, ng/mL, median (Q1–Q3)	33.8 (23.6–49.8)	48.5 (22.7–92.2)	27.6 (23.1–41.2)	0.21
	CK-MB, U/L, median (Q1–Q3)	0.8 (0.5–1.3)	1.3 (0.5–2.9)	0.7 (0.5–1.0)	0.13
	CRP, mg/L, median (Q1–Q3)	3.7 (2.1–5.7)	5.7 (4.7–9.7)	2.1 (1.5–2.3)	<0.01*
	Creatinine, mg/dL, median (Q1–Q3)	65.2 (56.6–79.4)	66.1 (62.0–114.1)	58.2 (50.8–74.5)	0.06
	D-dimer, µg/mL, median (Q1–Q3)	0.4 (0.2–3.2)	2.9 (0.7–4.8)	0.2 (0.2–0.5)	0.03*
	ALT, IU/L, median (Q1–Q3)	25.0 (14.0–40.0)	26.0 (13.0–36.5)	21.5 (14.8–53.8)	0.82
	Albumin, g/L, median (Q1–Q3)	45.3 (40.1–46.5)	43.6 (35.4–45.6)	45.9 (45.2–48.1)	0.02*
Echocardiography on admission					
	LVEDD, mm, mean ± SD	47.6 ± 4.2	50.3 ± 2.7	45.2 ± 3.8	0.01*
	LVEF, %, mean ± SD	64.3 ± 4.9	61.3 ± 4.4	63.0 ± 3.7	<0.01*

AVB, atrioventricular block; BMI, body mass index; WBC, white blood cell; hs-cTnI, high-sensitivity cardiac troponin I; NT-pro BNP, N-terminal pro b-type natriuretic peptide; MYO, myoglobulin; CK-MB, creatine kinase; CRP, C-reactive protein; ALT, alanine transaminase; LVEDD, left ventricular end diastolic diameter; LVEF, left ventricular ejection fraction; SD, standard deviation; ECG, electrocardiogram. * indicates statistically significant; COVID-19, coronavirus disease 2019.

### 3.2 Pacemaker Implantation and Follow-Ups

All 17 COVID-19-related AVB patients received dual-chamber pacemaker implantation. Atrial leads were all placed in the right atrial appendage, ventricular leads were implanted at the left bundle branch area (10 patients) and right ventricular septum (7 patients). No complication occurred during procedure. The intrinsic QRS duration was 112.8 ± 20.0 ms and the paced QRS duration was 134.6 ± 17.5 ms.

At implantation, sensed and paced atrioventricular intervals were programmed as 170 and 200 ms for standardization. The mean threshold, impendence, and sense of ventricular leads were 0.5 mV, 578.6 Ω, and 12.0 mV. The mean threshold, impendence, and sense of atrial leads were 0.7 mV, 656.6 Ω, and 2.8 mV. The median initial ventricular pacing (VP) ratio was 100.0% (Q1–Q3: 100.0–100.0). At follow-up, the median VP ratio remained 100.0% (Q1–Q3: 98.5–100.0). The mean threshold, impendence, and sense of ventricular leads were 0.7 mV, 549.6 Ω, and 14.9 mV. The mean threshold, impendence, and sense of atrial leads were 0.6 mV, 598.5 Ω, and 3.7 mV.

### 3.3 Data-Independent Acquisition Mass Spectrometry for Proteomic Profiling of COVID-19-Related AVB Sera

We obtained 8895 peptides in total, with an average number ranging from 5492 to 5815.2 in COVID-19-related AVB patients and controls. We quantified and mapped these peptides to corresponding protein sequences with the MaxQuant software package [12]. We identified 397 human proteins, with an average range ranging from 293 to 312.3.

**Fig. 2.**
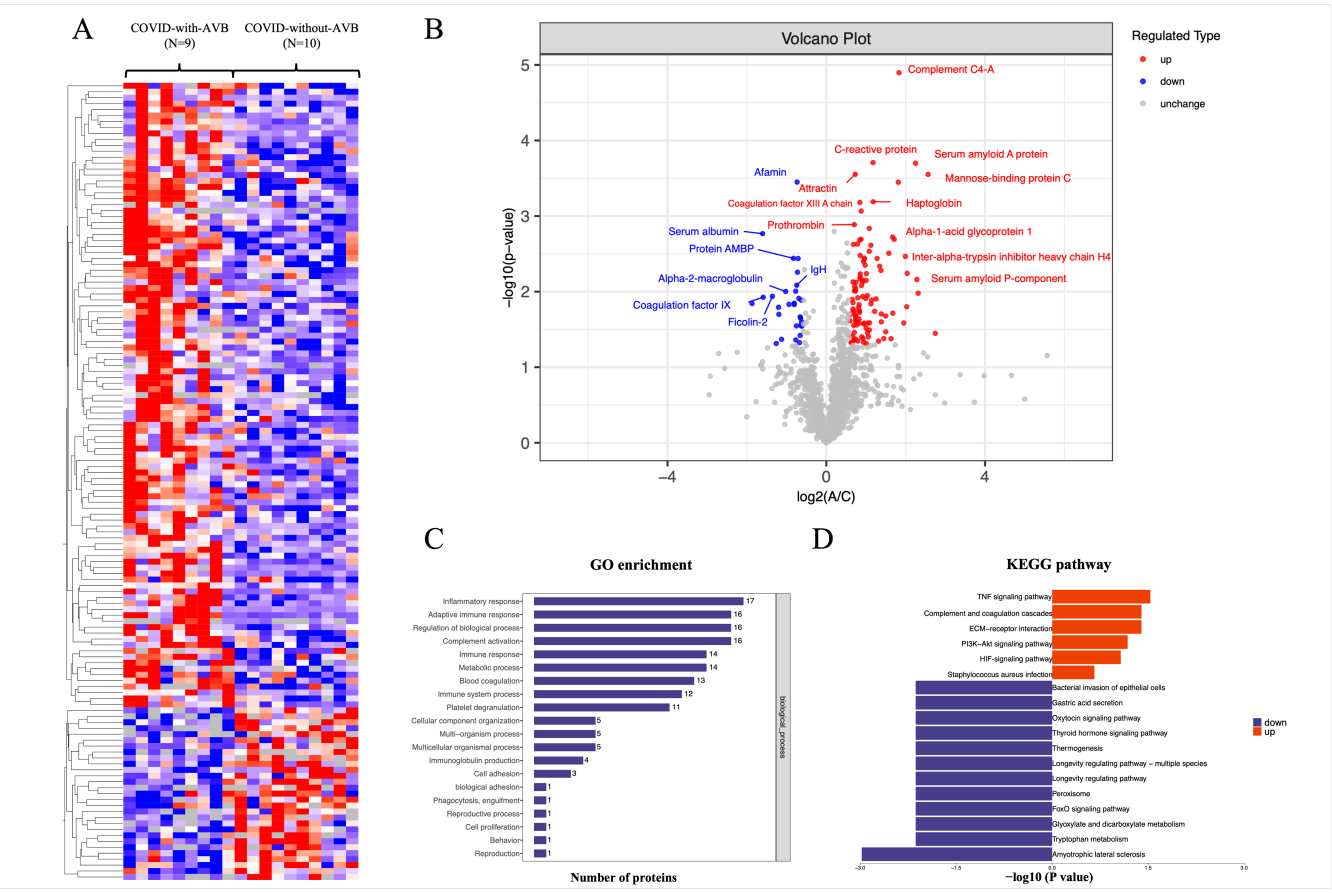
**Summary of comparative proteomics analysis of COVID-19-related AVB patients and controls.** (A) The heatmap of the quantified peptides and proteins in the 19 plasma samples. (B) The volcano map of the reserved proteins in the plasma samples. (C) GO-based enrichment analysis of differential proteins of the COVID-with-AVB group and COVID-without-AVB group. (D) KEGG-based enrichment analysis of differential proteins of the COVID-with-AVB group and COVID-without-AVB group. COVID-19, coronavirus disease 2019; AVB, atrioventricular block; GO, gene ontology; KEGG, kyoto encyclopedia of genes and genomes; Ig, immunoglobulin; TNF, tumor necrosis factor; ECM, extracellular matrix; HIF, hypoxia-inducible factor; AMBP, alpha-1-microglobulin/bikunin precursor; IgH, immunoglobulin heavy chain; PI3K-Akt, phosphatidylinositol 3-kinase; FoxO, forkhead box O.

To ensure the quality and reliability of the proteomic data, we analyzed the protein mutually found in >70% of samples. We performed multivariate normal imputation to impute the missing values. We performed the principal-component analysis in 20 samples using 397 quantified proteins and 233 proteins after imputation. We analyzed the reserved proteins using the hierarchical clustering method (Fig. 2A). We concluded from the visualized heatmap that several proteins are differentially expressed in different groups (Fig. 2B), indicating that single biomarkers could be found from the proteomic data.

### 3.4 Proteomic Changes of COVID-19-Related AVB Sera

We analyzed the difference in plasma proteins to explore the signature of COVID-19-related permanent AVB. The definition of significant fold changes (FC) was |log2(FC)| >0.5 with unpaired two-sided Welch’s *t*-test *p* < 0.05. We identified a total of 134 proteins differentially expressed in COVID-with the AVB group and COVID-without the AVB group. Those differentially expressed proteins were subjected to the Kyoto Encyclopedia of Genes and Genomes (KEGG) and Gene Ontology (GO) pathway enrichment analysis, which indicates those proteins were highly enriched in the inflammatory response and complement and coagulation cascades (Fig. 2C,D). Such results are consistent with the inflammatory process and abnormal immune response previously reported in COVID-19 patients.

The inflammatory response obtained the highest enrichment ratio scores in the KEGG and GO analyses between the COVID-with the AVB group and COVID-without the AVB group. Moreover, the proteins involved in these processes were substantially altered in three groups. These results were consistent with clinical data that the C-reactive protein showed a significant difference in the COVID-19-with the AVB group and COVID-19-without the AVB group. These findings indicated that coagulation cascades and platelet dysfunction are the consequence of COVID-19, and inflammatory disorder might contribute to the pathogenesis of COVID-19-related AVB.

### 3.5 The Alterations of Host Proteins are Associated with COVID-19-Related AVB

In addition to proteins related to the inflammatory response and complement and coagulation cascades, we also identified several alterations to host proteins. The plasma level of tetranectin, haptoglobin, neutrophil defensin 3, and fibrinogen beta chain were significantly elevated in the COVID-19-related AVB group. Tetranectin and haptoglobin are associated with platelet degranulation. Tetranectin enables calcium ion binding activity and cellular response to transforming growth factor beta stimulus related to the acute-phase response and viral receptor activity [13]. Haptoglobin functions to bind free plasma hemoglobin, which plays a role in modulating many aspects of the acute phase response and has antibacterial activity [14]. Neutrophil defensin 3 is a cytotoxic peptide of the innate immune system involved in reaction to bacteria, fungi, and viruses [15]. The Fibrinogen beta chain is the beta component of fibrinogen and may facilitate the immune response via innate and T-cell-mediated pathways [16]. These plasma proteins imply the potential activation of platelet degranulation and immune response to COVID-19 infection.

We identified that protein alpha-1-microglobulin/bikunin precursor (AMBP) and alpha-2-macroglobulin levels were substantially reduced in the COVID-19-with the AVB group. Protein AMBP is a complex glycoprotein that is an effector molecule in regulating inflammatory processes [17, 18]. Alpha-2-macroglobulin is a cytokine transporter and protease inhibitor of inflammatory cytokines and cascades [19, 20]. Therefore, the reduction of these anti-inflammatory proteins may intensify the inflammatory response.

## 4. Discussion

COVID-19 is a multisystem disease that predominantly influences the respiratory system. However, cardiovascular manifestations including myocardial infarction, myocarditis, and heart failure are not rare [21, 22, 23]. Patients with COVID-19 are at risk for certain arrhythmias, including heart rate–corrected QT interval (QTc) prolongation, supraventricular tachycardia, ventricular tachycardias, and conduction system diseases. Though the incidence of COVID-19-related conduction system diseases is lower than tachycardias, they have been demonstrated to be independent drivers of mortality and adverse events. By retrospectively analyzing 756 patients with COVID-19, McCullough *et al*. [24] reported atrial fibrillation and flutter occurred in 5.6%, atrioventricular block in 2.6%, and intraventricular conduction block in 11.8%. They concluded that a right bundle branch block or intraventricular block (odds ratio = 2.61, *p* = 0.002) increased the odds of mortality. Moreover, Pavri *et al*. [25] revealed that abnormal PR prolongation was associated with an increased risk of endotracheal intubation and death.

The prevalence of COVID-19-related AVB has yet to be well established. In patients hospitalized with COVID-19, studies have reported new-onset AVB occurred in 0.02–11.8% in different study populations [4, 26, 27]. Coromilas *et al*. [26] retrospectively analyzed patients hospitalized with COVID-19 infection worldwide, and reported the incidence of AVB is 0.02%. Among generalized hospitalized patients with COVID-19, Lao *et al*. [27] and Antwi-Amoabeng *et al*. [4] reported that the incidence of AVB was 3.6% and 11.8%. In symptomatic COVID-19 cases, Kunal *et al*. [23] revealed 5 AVB cases (4.6%) in 109 patients. Our study retrospectively included 72 hospitalized patients with AVB from November 2022 to March 2023 and reported 17 patients with new-onset AVB associated with COVID-19.

The molecular insights for the pathogenesis of COVID-19-related AVB remain to be clarified. Several hypotheses have been proposed, including cytokine storm, direct viral injury, and hypoxia. SARS-CoV-2 infection can induce an increased systemic inflammatory response and cytokine storm manifested by an acute elevation of serum Interleukin-6 (IL-6), Interleukin-1 (IL-1), tumor necrosis factor-α (TNF-α), and transforming growth factor- β1 (TGF-β1). Accumulating data reveal that inflammatory cytokines can affect the intrinsic conduction system by means of directly affecting the cardiomyocytes, expression, and function of ion channel proteins and connexins [6]. IL-6, IL-1, and TNF-α have been shown to described that modulate the expression of T-type calcium channels (${\mathrm{Ca}}_{\text{V}}$3.1 and ${\mathrm{Ca}}_{\text{V}}$3.2) and L-type calcium channels (${\mathrm{Ca}}_{\text{V}}$1.2 and ${\mathrm{Ca}}_{\text{V}}$1.3) and subsequently slow the atrioventricular conduction [28]. Lazzerini *et al*. [6] demonstrated that systemic inflammation can cause acute atrioventricular delay, and IL-6 can downregulate the cardiac connexin43 that affects conduction velocity in animal models. Recent research indicates that activation of the transforming growth factor superfamily can cause deleterious effects in multiple organ systems in patients with COVID-19. Wang *et al*. [29] observed that serum levels of TGF-β1 were significantly increased in COVID-19 patients. TGF-β1 is a multifunctional cytokine that plays a crucial role in cardiac arrhythmogenicity. TGF-β1 plays a pivotal role in regulating T-box-transcription factor T-Box Transcription Factor 3 (TBX3), which controls the embryogenic development of the human cardiac conduction system [30]. Moreover, TGF-β1 is reported to modulate electrophysiological properties of the atrioventricular junction via canonical Wnt signaling [31].

Comparative proteomics analysis revealed altered proteins that are highly enriched in the inflammatory response. For instance, plasma levels of serum amyloid A protein, C-reactive protein, Alpha-1-acid glycoprotein 1, mannose-binding protein C, haptoglobin, and attractin were significantly elevated in the COVID-19-related AVB group. Among them, serum amyloid A protein, C-reactive protein, Alpha-1-acid glycoprotein 1, and mannose-binding protein C are acute-phase proteins that are closely related to infection and inflammation. C-reactive protein and serum amyloid A protein were also identified to be significantly elevated in previously reported COVID-19 patients [32]. C-reactive protein and serum amyloid A protein are major acute phase proteins in controlling and propagating the acute phase response. Emerging evidence indicates systemic inflammation can negatively influence autonomic function and atrioventricular conduction. Haarala *et al*. [33] demonstrated that reduced heart rate variability is associated with elevated C-reactive protein and serum amyloid A protein. Lazzerini *et al*. [28] found that atrioventricular conduction indices are increased with elevated C-reactive protein levels and are rapidly normalized in association with the reduction of inflammatory markers. Such findings corresponded with the clinical fingdings and lab results, which indicated AVB may be the sequelae of myocarditis. Blood tests from myocarditis patients often show elevated cardiac enzymes as troponin and inflammatory markers as C-reactive protein, lactate, and procalcitonin [2]. The median level of hs-cTnI and NT-pro BNP of COVID-19-relatedAVB is significantly higher than controls, which is supportive for diagnosing COVID-19-related myocarditis. Whether those acute-phase proteins are pivotal to the development of AVB in COVID-19 patient needs to be further elucidated.

SARS-CoV-2 may cause direct injury to the cardiac conduction system via the angiotensin-converting enzyme 2 (ACE2) receptor. Cardiomyocyte entry of SARS-CoV-2 may depend on binding the viral spike proteins to ACE2 receptors and on S protein priming by proteases. ACE2 receptors are abundant in the kidney and heart. Moreover, evidence shows ACE2 receptors be located in the cardiac conduction system [34]. Han *et al*. [8] revealed SARS-CoV-2 can infect primary pacemaker cells in the heart and induce ferroptosis that lead to arrhythmias. Vaduganathan *et al*. [9] demonstrated that angiotensin II is accumulated via virus-associated down-regulation of ACE2, resulting in adverse myocardial remodeling and arrhythmogenicity.

The majority of the literature documented that COVID-19-related AVB were transient and self-limiting. Dagher *et al*. [35] reported 4 patients developing a transient high-degree AVB during hospitalization, with 1 patient being implanted with a temporary pacemaker. After 10–21 days from admission, all patients were discharged in sinus rhythm and a normal PR interval. The spontaneous resolution of COVID-19-related AVB reveals the nature of the viral infection. However, cases of permanent AVB patients requiring paroxysmal or permanent pacing have been well reported. In our study, 8 paroxysmal AVB with symptomatic manifestations of bradycardia had permanent pacemaker implantation. Sensed and paced atrioventricular intervals were programmed as 170 and 200 ms to stimulate intrinsic conduction. After a mean follow-up of 2.9 months, the median VP ratio was 99.8%, which reveals the potential long-term damage to the conduction system. Therefore, the long-term follow-up of symptomatic AVB patients with spontaneous resolution is necessary.

We successfully performed left bundle branch area pacing (LBBAP) and right ventricular septal paing (RVSP) in all COVID-19-related AVB patients. No procedural-related complication occurred. The pacing parameters, including threshold, sense, and impendence remained stable at follow-up. Such finding indicated LBBAP and RVSP are both safe and effective to treat COVID-19-related AVB.

## 5. Study Limitations

Our study has several limitations inherent to the design. The single-center nature of our study may potentially limit the sample size and introduce selection bias. Our study recruited hospitalized AVB patients associated with SARS-CoV-2 infection which lacks the confidence to demonstrate the causal relationship between AVB and COVID-19. However, based on Hill’s Criteria, we observed the temporal order of COVID-19 and the onset of AVB and carried out lab tests to exclude other potential viral causes besides SARS-CoV-2 in our cohort. Moreover, our study is consistent with other cross-sectional studies [4, 26, 27], case report [35], and review [5], which demonstrate the analogy and plausibility of AVB and COVID-19. Based on Han’s study [8], we believe it is reasonable to assume AVB may be the sequelae of SARS-CoV-2 infection. Additionally, most serum samples were collected from the patients in the late period of the disease, thus limiting the proteomic profiling of the acute phase. Our research also lacked a large independent cohort to validate the potential biomarkers. Despite these limitations, we believe our research uncovers important knowledge pertaining to COVID-19-related AVB. Further studies are necessary for further proteomic validation and the detailed pathogenesis of COVID-19-related AVB.

## 6. Conclusions

Our study depicts the clinical characteristics and concludes both LBBAP and RVSP are safe and effective to treat COVID-19-related AVB. We provides a valuable resource of proteomics profile in which several differential expressed proteins (Serum amyloid A protein, Tetranectin, Neutrophil defensin 3) enriched to inflammatory response, complement and coagulation cascades, and immune response. Our results sheds light on identifying the potential biomarkers and pathogenesis of COVID-19-related AVB.

## Data Availability

The data regarding personal information is subjected to legal restriction of Chinese legislation. The request to access the data can be granted or rejected by State Key Laboratory of Cardiovascular Disease, Fuwai Hospital, National Center for Cardiovascular Diseases, Chinese Academy of Medical Sciences and Peking Union Medical College.
